# Draft Genome Sequence of *Geobacillus* sp. LEMMY01, a Thermophilic Bacterium Isolated from the Site of a Burning Grass Pile

**DOI:** 10.1128/genomeA.00200-17

**Published:** 2017-05-11

**Authors:** Yuri Pinheiro Alves de Souza, Fábio Faria da Mota, Alexandre Soares Rosado

**Affiliations:** a Laboratório de Ecologia Molecular Microbiana (LEMM), Instituto de Microbiologia Paulo de Góes, Federal University of Rio de Janeiro, Rio de Janeiro, Brazil; b Laboratório de Biologia Computacional e Sistemas, Instituto Oswaldo Cruz, FIOCRUZ, Rio de Janeiro, Brazil

## Abstract

We report here the 3,586,065-bp draft genome of *Geobacillus* sp. LEMMY01, which was isolated (axenic culture) from a thermophilic chemolitoautotrophic consortium obtained from the site of a burning grass pile. The genome contains biosynthetic gene clusters coding for secondary metabolites, such as terpene and lantipeptide, confirming the biotechnological potential of this strain.

## GENOME ANNOUNCEMENT

*Geobacillus* spp. are rod shaped, Gram-positive, aerobic or facultatively anaerobic, endospore-forming, thermophilic microbes. The majority of isolated *Geobacillus* spp. can grow over a range of 45 to 75°C and seem to be ubiquitous (1). These characteristics make members of this genus highly suitable for biotechnological applications, as sources for thermostable enzymes with great potential for biocatalysis and production of potential novel secondary metabolites (2).

We sequenced the draft genome of *Geobacillus* sp. strain LEMMY01, which was isolated in axenic culture from a thermophilic chemolitoautotrophic consortium obtained from a soil sample collected from the site of a burning grass pile located in Seropédica, Rio de Janeiro, Brazil (our unpublished results). This consortium was obtained in a mineral medium, N-FIX (3), and was further inoculated on plates with solid GYM medium (DSMZ 65) and incubated at 55°C. After 48 h, isolated colonies were grown in liquid GYM medium. The DNA was extracted using a FastDNA SPIN kit for soil (MpBio) and quantified using a QUBIT fluorimeter (Thermo Fisher Scientific).

The Nextera XT DNA library kit was used to build paired-end 250-bp libraries, which were sequenced on an Illumina MiSeq platform. Assembly was performed by MR DNA (Shallowater, TX, USA) using NGen version 12 from DNAstar, and annotation was performed using the NCBI Prokaryotic Genome Annotation Pipeline (PGAP). The draft assembly of *Geobacillus* sp. LEMMY01 is 3,586,065 bp in length (244× coverage, 51.91% GC content) in 76 contigs with an *N*_50_ of 81.839 bp. The presence of antibiotic resistance genes was checked using Resfinder (4) with no results. Genes related to the production of terpenes and lantipeptide were detected using antiSMASH (5), which confirms the biotechnological potential of this strain.

### Accession number(s).

This whole-genome shotgun project has been deposited at DDBJ/ENA/GenBank under the accession number MVKA00000000. The version described in this paper is the first version, MVKA01000000.
